# Current research and future prospects of immunonutrition in gastrointestinal malignancies

**DOI:** 10.3389/fimmu.2024.1420415

**Published:** 2024-09-06

**Authors:** Xiaoyan Ma, Beibei Pei, Na Wu, Chen Wang, Yanling Yu, Wenhui Yang

**Affiliations:** ^1^ Third Hospital of Shanxi Medical University, Shanxi Bethune Hospital, Shanxi Academy of Medical Sciences, Tongji Shanxi Hospital, Taiyuan, China; ^2^ Shanxi Province Cancer Hospital, Affiliated Cancer Hospital of Shanxi Medical University, Taiyuan, China

**Keywords:** gastrointestinal malignancy, immunonutrition, arginine, glutamine, omega-3 polyunsaturated fatty acids (ω-3PUFAs)

## Abstract

Immune nutrition, as an integral component of nutritional support therapy, has garnered significant attention and research in the treatment of gastrointestinal malignancies. Recent advancements in nutritional formulas containing components such as glutamine, omega-3 polyunsaturated fatty acids, and arginine have led to the development of what is now termed immune nutrition or pharmacological nutrition. These formulations go beyond traditional nutritional support, functioning more like nutritional supplements with pharmacological effects. Patients with gastrointestinal malignancies often experience malnutrition and metabolic disturbances, resulting in immune dysfunction, cytokine dysregulation, and endocrine abnormalities. These issues can compromise intestinal mucosal barrier function, affecting the efficacy and prognosis of anticancer therapies. Recent studies indicate that immune nutrition can modulate specific mechanisms involved in various immune and inflammatory pathways, thereby improving patients’ immune status and treatment outcomes. While optimal patient selection, dosing, and timing of immune nutrition are still under investigation, its potential applications in oncology are promising. This article aims to analyze the existing evidence regarding the therapeutic benefits of immune nutrition in gastrointestinal malignancies, offering insights into its clinical standardization and application.

## 1 Introduction

**Figure 1:**
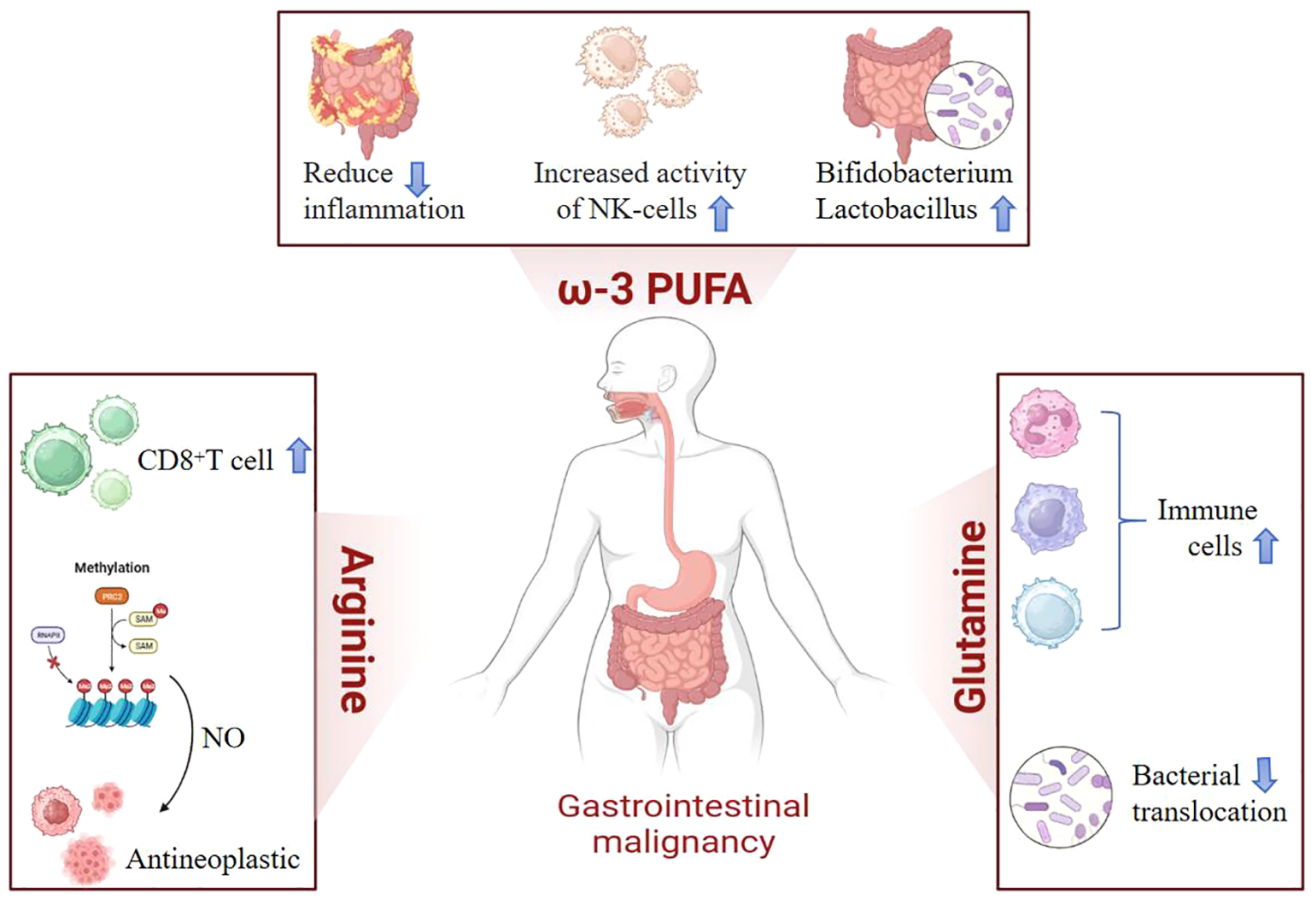
In clinical practice, commonly used immunonutrients include arginine, omega-3 polyunsaturated fatty acids (omega-3 PUFA), and glutamine. Arginine promotes the proliferation of T lymphocytes and enhances immune responses. Its metabolite, nitric oxide (NO), acts as a free radical that maintains microcirculation and eliminates microorganisms. Not only does it possess anti-tumor properties, but it also enhances the sensitivity of chemotherapy by regulating tumor vascular function and other aspects. Omega-3 polyunsaturated fatty acids regulate the body’s inflammatory responses, increase natural killer cell activity, and promote the growth of beneficial intestinal flora. Glutamine plays a positive role in protecting and maintaining the normal function of the gastrointestinal mucosa, increasing the total number of lymphocytes, and preventing bacterial translocation.

Globally, gastrointestinal malignancies are among the top five most common cancers (1). Throughout the course of these diseases, patients often experience malnutrition, metabolic abnormalities, immune imbalances, and inflammatory responses. Notably, malnutrition is an independent predictive factor for postoperative morbidity and mortality (2). Nutritional support is widely used for patients with gastrointestinal tumors, and immunonutrition, which includes enhanced nutritional components, has emerged as a promising approach in cancer therapy. The key immunonutrients used in these interventions include glutamine, arginine, omega-3 polyunsaturated fatty acids (omega-3 PUFA), sulfur-containing amino acids, antioxidants, and nucleotides, either alone or in various combinations (3). Understanding the interactions between immune cells, tumor cells, and cancer treatments can help clinicians better utilize the body’s immune response against cancer. In the early stages of tumor development, immune effector cells can recognize and destroy malignant cells. However, as the tumor progresses, a balance is often achieved between malignant cells and the immune response within the tumor lesion, allowing tumor cells to evade immune system detection and promote tumor growth and metastasis (4). Proper nutrition is crucial for maintaining immune system function. Vitamins, micronutrients, omega-3 PUFA, and other essential nutrients play significant and complementary roles in supporting the immune response. Insufficient intake of these nutrients can lead to decreased resistance to infections and an increased disease burden (5). Immunonutrition can modulate specific mechanisms involved in various immune and inflammatory pathways (**Figure 1**). It has been studied in the context of elective surgeries, where it has been shown to reduce the risk of complications and mortality, as well as shorten hospital stays. Furthermore, immunonutrition appears to alleviate adverse reactions in patients undergoing radiotherapy and chemotherapy (6).

## 2 Mechanism of malnutrition in patients with gastrointestinal malignancies

Malnutrition, defined as a deficiency in energy, proteins, and other essential nutrients, can significantly impair bodily functions and worsen clinical outcomes. Patients with malignancies often experience a state of hypermetabolism, wherein tumor cells demonstrate the “Warburg effect.” This effect describes a preference for glycolysis over the tricarboxylic acid cycle for energy production, even when mitochondrial function is fully intact, thereby facilitating tumor growth. The glycolytic rate in malignant tumors can be 10 to 100 times higher than the rate of complete glucose oxidation in normal tissue mitochondria (7). Furthermore, under normal circumstances, cellular nutrient uptake is typically regulated by growth factor stimulation to prevent excessive proliferation when nutrient availability surpasses the requirements for cellular division. However, cancer cells often acquire oncogenic mutations that enable them to bypass this growth factor dependency, resulting in increased nutrient uptake and metabolism (8).

Compared to other types of cancer, patients with gastrointestinal malignancies have a higher prevalence of malnutrition. It is estimated that up to 80% of these patients are at risk of malnutrition at the time of diagnosis (9). This high prevalence may be attributed to the primary tumor’s impact on the gastrointestinal tract, causing symptoms such as poor appetite, gastrointestinal obstruction, dysphagia, early satiety, taste changes, etc. (10). The treatment of malignant tumors can also indirectly increase the risk of malnutrition. For instance, surgical interventions, along with associated trauma from surgery and anesthesia, can exacerbate malnutrition. If a patient’s nutritional status is not promptly assessed and addressed, the incidence of postoperative complications may rise significantly. This can lead to suboptimal surgical outcomes, an increased likelihood of infectious complications, delayed wound healing, and prolonged length of hospital stay (LOS) (11). Radiotherapy can adversely affect the function of normal tissues and organs within the target area, compromising the health and function of the gastrointestinal mucosa and potentially causing inflammation or ulcers (12, 13). These effects can negatively impact oral intake (14). Similarly, current chemotherapy regimens often do not differentiate between cancer cells and normal cells, such as gastrointestinal epithelial cells, leading to a range of gastrointestinal-related side effects (15). Additionally, chemotherapy drugs can impose a burden on organs like the liver and kidneys (16). Common digestive system-related side effects of radiotherapy and chemotherapy include nausea, vomiting, oral ulcers, diarrhea, and loss of appetite (17). These side effects not only directly reduce appetite and food intake but also contribute to nutrient loss and depletion.

## 3 Immunomodulatory effects of main immunomodulatory nutritional preparations

### 3.1 Glutamine

Glutamine, the most abundant amino acid in the body, is crucial for maintaining immune function, nitrogen balance, intestinal integrity, and combating infections (18). In a mouse model of gastric cancer, oral glutamine administration decreased tissue levels of glutathione while increasing plasma glutathione levels. This treatment also enhanced T lymphocyte proliferation, increased NK cell activity, significantly suppressed TNF-α secretion, and promoted interleukin-2 (IL-2) secretion, thereby modulating immune function *in vivo* (19). Glutamine also plays a role in intracellular signaling by increasing the expression of heat shock proteins, inhibiting apoptosis, and reducing inflammatory responses (20). Current evidence indicates that during catabolic states, decreased glutamine levels may increase the risk of secondary infections, prolong recovery time, and elevate mortality rates. Supplementation with glutamine in critically ill or surgical patients has been shown to reduce the incidence of hospital-acquired infections and complications, as well as shorten LOS (21). Additionally, an oligomeric diet rich in glutamine has demonstrated protective effects against gastrointestinal toxicity associated with chemotherapy and radiotherapy in patients with rectal cancer (22). However, it is essential to maintain a balanced concentration of glutamine to achieve effective anti-tumor effects. Colorectal cancer has demonstrated a notable reliance on glutamine, as tumor cells utilize it as an energy and nutrient source, thereby facilitating tumor growth and metastasis. Notably, the degree of glutamine dependence and the response characteristics can differ among various colorectal cancer cell lines, highlighting the need for further research to deepen our understanding of these mechanisms (23). Currently, there are significant variations in the proportion and concentration of glutamine in enteral nutrition formulations as indicated on domestic and international drug labels and in clinical practice. A randomized controlled trial evaluated the impact of different proportions of alanine-glutamine (Ala-Gln) in parenteral nutrition on postoperative nutrition, liver and kidney function, and immune status in patients with gastrointestinal cancer. The results showed no significant differences in postoperative nutrition, liver and kidney function, or recovery among the different Ala-Gln proportions. However, in terms of immune modulation, a higher proportion of Ala-Gln (30% of total amino acids in parenteral nutrition) appeared to have superior effects on T lymphocyte immune modulation (24).

### 3.2 Arginine

Arginine, a non-essential amino acid under normal circumstances, becomes conditionally essential during critical illness (25). Known as a “supplement for T cells,” arginine can promote T lymphocyte proliferation and enhance immune responses (26). Previous evidence has demonstrated that dietary supplements containing arginine can increase protein and collagen deposition in experimental wounds and enhance T-cell activity (27). Additionally, Fu et al. found that arginine can regulate postoperative inflammatory responses and immune function by increasing the concentration of immunoglobulin IgM (28). Arginine plays a role in activating anti-tumor immunity and regulating the efficacy of immunotherapy (29), primarily through two metabolic pathways involving arginase and nitric oxide (NO), which are crucial for immune regulation. NO, a metabolic product in the body, is a free radical that helps maintain microcirculation and eliminate microorganisms. It not only exhibits anti-tumor effects but also enhances chemotherapy sensitivity by regulating tumor vascular function, among other effects (30). However, clinicians should exercise caution when supplementing arginine, as excessive NO production induced by inducible nitric oxide synthase can lead to tissue damage. In cases of severe inflammation, arginine supplementation may exacerbate the inflammatory condition. Therefore, the use of arginine should be carefully considered in clinical applications, with attention to optimal dosage, timing, and patient conditions (31).

### 3.3 Omega-3 polyunsaturated fatty acids

ω-3 PUFAs are essential fatty acids rich in alpha-linolenic acid (ALA), docosahexaenoic acid (DHA), and eicosapentaenoic acid (EPA), primarily found in fish oil. These unsaturated fatty acids can be obtained through dietary intake. The functions of ω-3 PUFAs include (1) Regulating inflammatory response: ω-3 PUFAs play a crucial role in regulating various biological functions, including inflammation and immune responses (32). EPA can significantly reduce the concentration of inflammatory cytokines, such as IL-6 and IL-10, aiding in the resolution of inflammation (33). A controlled trial involving 698 patients demonstrated that fish oil supplementation had a positive effect on inflammatory markers in gastric cancer patients undergoing surgery (34). Another study showed that postoperative application of ω-3 PUFAs could enhance NK cell activity, helping maintain the immune response at a moderate sensitivity level, possibly related to the methylation status of the TNF-α gene promoter (2, 35). Maintaining intestinal mucosal barrier function: Supplementing with ω-3 PUFAs can regulate the gut microbiota, increasing the diversity and abundance of beneficial bacteria (36). A randomized controlled trial observed that a diet rich in ω-3 PUFAs significantly increased the abundance of several short-chain fatty acid-producing genera, including Bifidobacterium, Roseobacterium, and Lactobacillus, and that this effect was reversible (37). ω-3 PUFAs also protect the intestinal epithelial barrier and the integrity of the gut microbiota composition, reducing bacterial translocation and preventing postoperative complications such as intestinal leakage syndrome 38). Cancer prevention: Nandi SK et al. has demonstrated that ω-3 PUFAs play a significant role in cancer prevention, demonstrating potent anti-proliferative effects. They are considered safe, well-tolerated, non-toxic, and cost-effective nutritional supplements (39). Research has shown that components like DHA and EPA can induce apoptosis in gastric cancer cells and arrest the cell cycle at the G0/G1 phase. When combined with chemotherapy drugs, they exhibit synergistic effects, ultimately inhibiting the proliferation of gastric cancer cells (40).

## 4 Clinical application of immunonutrition in gastrointestinal surgery

Recent advances in the surgical treatment of gastrointestinal malignancies have improved patient outcomes. However, surgery can compromise the structural and functional integrity of the gastrointestinal tract, trigger systemic inflammatory stress responses, and lead to various endocrine, immune, and hematological effects. Postoperative complications remain common. Immunonutrition has been widely adopted in clinical practice for patients with gastrointestinal malignancies, offering significant improvements in nutritional status (41, 42). Additionally, it has demonstrated positive benefits in several other aspects.

### 4.1 Reduce overall complication

The use of immunonutrition in surgical settings—preoperatively, postoperatively, and in combination—has consistently been associated with a reduction in overall complication rates (43).

According to a meta-analysis, early preoperative immune nutrition significantly reduced the incidence of postoperative abdominal surgical site infections compared to the absence of oral nutritional supplements (44–46). In a study conducted by Yu et al. (47), 112 patients with gastric cancer cachexia were randomly assigned to two groups at a 1:1 ratio. One group received enteral immune nutrition (IN) support, including ω-3 polyunsaturated fatty acids, L-arginine, and nucleotides, providing approximately 1063 kcal. The other group received standard enteral nutrition (SEN), which was isonitrogenous and isocaloric. Nutritional support was provided from the 7th to the 1st day before surgery. The results indicated that the incidence of postoperative infectious complications and the duration of antibiotic use were significantly lower in the IN group compared to the SEN group. However, not all studies have reached similar conclusions. Some research has shown no clear association between preoperative immunonutrition and a reduction in infectious complications in patients undergoing colon cancer surgery (48). Thus, the routine use of immunonutrition before abdominal surgery requires further research to evaluate its efficacy.

According to current guidelines, immunonutrition is recommended for patients with perioperative gastrointestinal malignancies to improve prognosis (49). A study involving 3,375 colorectal cancer (CRC) patients undergoing elective rectal surgery found that the rates of severe adverse events were 6.8% in patients receiving immunonutrition and 8.3% in those not receiving it. Additionally, the rates of prolonged hospital stays were 13.8% and 17.3%, respectively (50). While these findings indicate some benefits, the effect may be limited, potentially due to variations in patient compliance. The most recent observational retrospective cohort study, which included 134 patients who received nutritional support for an average of 10 days before and after surgery, reached similar conclusions. Compared to the standard nutrition group, the IN group experienced a 34% reduction in hospital stay duration, a 70.1% decrease in infectious complications, and varying degrees of reduction in other postoperative complications, such as intestinal obstruction, wound dehiscence, transfusion risk, pleural effusion, and acute kidney injury (51).

Studies providing enteral immunonutrition to patients solely after surgery have shown that the incidence of postoperative infection complications (7.4%) is significantly lower than that in the control group (20%). Additionally, the LOS was notably reduced (12.7 ± 2.3 days). However, these studies did not observe a significant impact on mortality (52). Early postoperative supplementation with formulations containing arginine, omega-3 PUFAs, and RNA has been demonstrated to increase hydroxyproline synthesis, thereby enhancing surgical wound healing in patients undergoing gastrectomy for gastric cancer (53). A meta-analysis estimated that immunonutrition interventions reduced the risk of infection complications by approximately 30% (54). Moreover, from a cost perspective, providing immunonutrition to patients undergoing elective surgery for gastrointestinal cancer is considered a cost-effective intervention (55).

### 4.2 Maintain intestinal barrier function and reduce inflammation

In a murine sepsis model, serum levels of TNF-α, IL-1β, and lactate were significantly higher in the control group compared to the immunonutrition group. Additionally, the colonic mucosal layer was noticeably thinner in the control group than in the immunonutrition group, suggesting a significant enhancement of intestinal mucosal barrier function by immunonutrients in septic mice (56). Building upon these animal model findings, researchers are investigating the practicality and potential application of these effects in clinical settings. A study assessing the impact of enteral immunonutritional support on postoperative immune function and intestinal mucosal barrier function following radical gastrectomy revealed that on postoperative day 3, levels of diamine oxidase, D-lactate, and endotoxin were notably lower in the immunonutrition group relative to the control group. These differences remained significant by postoperative day 7, with the immunonutrition group also showing significantly faster recovery times in terms of first flatus and defecation (57). In another trial involving patients undergoing radical surgery for CRC, perioperative enteral nutrition support was provided, with the addition of glutamine injection and probiotic quadruple strain tablets to the study group compared to the control group. Results demonstrated that on postoperative day 7, lactate, CRP, TNF-α, and IL-6 levels were reduced in the study group relative to the control group. Furthermore, CD4+ and CD4+/CD8+ levels were higher in the study group than in the control group (58).

Some scholars have conducted studies to assess the benefits of incorporating single-variety nutritional preparations for patients. In a clinical trial by Yang et al., the effects of EN combined with ω-3 PUFA on immune-related indicators and early recovery in gastric cancer patients were investigated. The study revealed that the immune and nutritional parameters of the ω-3 PUFA group were significantly higher than those of the control group. Additionally, inflammatory markers were lower in the ω-3 PUFA group, and these patients experienced earlier first flatus and defecation times compared to the control group (59). These results indicate that immunonutrition therapy can improve intestinal mucosal barrier function, reduce inflammatory responses, and enhance immune function in patients undergoing gastrointestinal surgery. These findings provide valuable evidence supporting the efficacy of immunonutrition in gastrointestinal surgery patients and pave the way for future prospective studies in this population.

## 5 Application of immunonutrition in radiotherapy and chemotherapy of gastrointestinal malignancies

### 5.1 Alleviating side effects of chemoradiotherapy

Chemotherapy and radiation therapy can cause indiscriminate damage to both normal and tumor cells, affecting metabolic processes and disrupting the gastrointestinal mucosal barrier. This disruption can lead to changes in the distribution of beneficial bacteria, such as Lactobacillus and Bifidobacterium, while increasing the presence of bacteria like Escherichia coli and Staphylococcus aureus. These changes result in intestinal dysfunction and side effects such as nausea, vomiting, diarrhea, mucositis, intestinal infections, and weight loss. Glutamine plays a crucial role in promoting the synthesis of glutathione and facilitating the regeneration of gastrointestinal epithelial cells, thus preserving the integrity of the mucosal barrier and reducing bacterial translocation. It helps alleviate the severity of diarrhea post-chemotherapy and radiotherapy, facilitates normal nutrient absorption, and aids in weight recovery (28, 48). Research conducted by Sun et al. demonstrated that supplementing glutamine as part of nutritional therapy can increase mucosal cell uptake of glutamine, reduce symptoms of mucosal pain and ulcers associated with gastrointestinal radiation therapy, and promote mucosal healing (60). Recent studies have reported that glutamine supplementation can reduce the inflammatory response in cancer patients. Specifically, enteral glutamine supplements have been shown to exhibit anti-inflammatory activity and reduce hormonal stress responses in rectal cancer patients receiving preoperative chemoradiotherapy, compared to placebo treatment (61). Another double-blind, randomized, controlled trial provided evidence that glutamine decreases the inflammatory response and mitigates changes in the autophagy machinery in patients receiving abdominal radiotherapy (62). Additionally, chemotherapy and radiation therapy can significantly impact the hematopoietic system, with bone marrow suppression being one of the common side effects. Severe bone marrow suppression increases the risk of infection and may even necessitate treatment interruption. A study by IMJAI et al. evaluated the effect of immunonutrient supplementation on related toxicities in patients undergoing chemotherapy and radiation therapy (63). The results demonstrated a notable reduction in the incidence of severe hematologic toxicity in patients supplemented with arginine, glutamine, and fish oil during concurrent chemoradiotherapy.

### 5.2 Improve the immune function of patients, synergistically enhance the efficacy of chemoradiotherapy

One of the significant challenges in chemotherapy and radiation therapy is ensuring that patients complete the planned cycles and doses without experiencing dose-limiting toxicities. This challenge has led researchers to investigate whether immunonutrition could bolster immune function, thereby improving patient tolerance and sensitivity to chemoradiotherapy and ultimately enhancing treatment efficacy. However, the reported outcomes of these interventions have varied, indicating the need for further research to clarify their potential benefits (64).

Mehmet Onur Gül et al. (65) investigated the impact of immunonutrition on tumor-infiltrating T lymphocytes and regulatory T cells in rectal cancer patients undergoing neoadjuvant chemoradiotherapy. Compared to the standard nutrition group, patients receiving immunonutrition before surgery exhibited a noteworthy increase in the CD4+/CD8+ ratio in resected tissues. This finding suggests that immunonutrition can effectively modulate the inflammatory stress response and immune function in rectal cancer patients undergoing neoadjuvant chemoradiotherapy, highlighting the potential benefits of this intervention. However, further research is essential to elucidate the underlying mechanisms. In a randomized controlled trial, Lamiss et al. explored the effects of ω-3 PUFA supplementation during neoadjuvant FLOT chemotherapy in individuals with locally advanced gastric cancer. The study revealed that ω-3 PUFA supplementation significantly mitigated the adverse effects of chemotherapy, including nausea, vomiting, fatigue, diarrhea, and weight loss, leading to reduced treatment interruptions and improved feasibility of curative surgery (66). This aligns with previous literature findings. LS174T cells have been identified as a model for CRC initiating cells displaying stem cell-like properties. Sam et al. assessed the impact of ω-3 PUFAs on LS174T cells and observed that ω-3 PUFAs hindered tumor cell growth by targeting survivin mRNA expression in these cells, demonstrating a dose-dependent relationship between ω-3 PUFAs concentration and its effects on tumor cells (67).

In a randomized, double-blind, placebo-controlled study with 73 preoperative chemoradiotherapy rectal cancer patients, continuous supplementation of 30g/day of glutamine over 5 weeks resulted in a significant reduction in IL-6 and cortisol levels, indicating the anti-inflammatory properties of glutamine within the intestines (61). Contrastingly, another study found that a daily dose of 13.5 grams of glutamine did not outperform the control group in preventing grade 3-4 non-hematological toxicity induced by chemotherapy in gastrointestinal cancer patients. The study’s discussion suggested that this lack of efficacy may be attributed to the suboptimal dosage of glutamine required for toxicity reduction (68). Arginine, as a vital component of immunonutrition, has demonstrated the ability to impede tumor cell growth by triggering caspase 8-mediated apoptosis, thereby augmenting the effectiveness of radiotherapy and chemotherapy (69). Reviewing the existing data supports arginine’s potential as a radio-protective, radio-alleviating, and radio-sensitizing agent (70).

While a specific immunonutritional regimen has shown promise in mitigating the side effects linked to radiotherapy and chemotherapy, the current evidence regarding the impact of immunonutrition on infectious complications or immune-related biomarkers in cancer patients undergoing chemotherapy remains limited (71). Given the lack of robust scientific support, individualized multidisciplinary approaches may offer valuable benefits tailored to each patient’s unique needs.

## 6 Summary and prospect

In conclusion, the current limited evidence supports the beneficial role of combined immunonutrition in treating gastrointestinal malignancies. This integrative approach not only enhances nutritional status but also modulates the host immune system and regulates inflammatory responses. Nonetheless, there is a lack of specific guidelines for immunonutrition therapy in gastrointestinal cancer patients, both nationally and globally. The existing guidelines and expert opinions offer fragmented and varied recommendations regarding immunonutrition supplementation. Large-scale, long-term prospective studies are essential to validate the enduring advantages of immunonutrition in combating tumors. In modern healthcare, effective implementation of immunonutrition strategies demands meticulous assessment, encompassing optimal nutrient combinations, timing of administration, suitable patient cohorts, and treatment duration. Recent advancements in immune therapies are reshaping the cancer treatment landscape, underscoring the challenge of seamlessly integrating nutrition support with immunotherapy to uphold patients’ immunonutritional well-being. Subsequent research should delve deeper into the interplay between immunonutrition and gastrointestinal malignancy treatment, tailoring personalized nutrition intervention plans to utilize the body’s immune response against cancer.
